# Artificially Expanded Genetic Information Systems for New Aptamer Technologies

**DOI:** 10.3390/biomedicines6020053

**Published:** 2018-05-09

**Authors:** Elisa Biondi, Steven A. Benner

**Affiliations:** 1 Foundation for Applied Molecular Evolution, Alachua, FL 32615, USA; sbenner@ffame.org; 2 Firebird Biomolecular Sciences, LLC, Alachua, FL 32615, USA

**Keywords:** expanded alphabet, AEGIS, aptamers, in vitro selection

## Abstract

Directed evolution was first applied to diverse libraries of DNA and RNA molecules a quarter century ago in the hope of gaining technology that would allow the creation of receptors, ligands, and catalysts on demand. Despite isolated successes, the outputs of this technology have been somewhat disappointing, perhaps because the four building blocks of standard DNA and RNA have too little functionality to have versatile binding properties, and offer too little information density to fold unambiguously. This review covers the recent literature that seeks to create an improved platform to support laboratory Darwinism, one based on an artificially expanded genetic information system (AEGIS) that adds independently replicating nucleotide “letters” to the evolving “alphabet”.

## 1. Introduction

More than a quarter century ago, Larry Gold, Jack Szostak, Gerald Joyce, Andrew Ellington, and others offered the promise of a process that would rapidly create receptors, ligands, and catalysts on demand [1,2]. All that was necessary was to create a library of nucleic acids, subject that library to processes by which species would be extracted that had some (and possibly low amounts) of the binding or catalytic activity desired, copy these “survivors” by PCR, possibly introducing mutations from species that had a low level of the desired activity, allowing improvement by locally searching the sequence space around such an individual, and repeating this process of selection/amplification until DNA or RNA binders or enzymes emerged with specifications that were adequate for the desired application. 

This hope was greatly magnified by the view that life on Earth itself had experienced an episode of natural history where nucleic acids (likely RNA) were the only genetically encoded compounds of biological catalysis, performing many of the roles that are now performed by proteins [3,4]. The existence of protein biosynthesis machinery (the ribosome) that was RNA in its catalytic part reinforced this enthusiasm [5]. Likewise, the role of RNA fragments in cofactors with a wide distribution across the Terran biosphere that makes their antiquity likely, further leads to the apparent reasonableness of this “RNA world” model.

Unfortunately, a quarter century on, this hope has not been realized as comprehensively as had been hoped. For sure, numerous binding molecules have emerged from selection applied to both DNA and RNA libraries [6,7,8]. These “aptamers” have seen use in research and, to a limited extent, medicine [9]. Some of these successes have been reviewed in this special edition [10,11,12,13,14,15]. 

However, overall, the performance of nucleic acid aptamers has not rivaled the performance of other binding molecules that can be generated with respect to specific targets [16,17,18,19,20,21,22,23,24,25,26]. Most notable among these, of course, are antibodies raised against antigens in living animals. Over the same time period, antibody replacements that are still proteins, but are created by diversity science that does not involve animals, have also become quite preferred in the biological community, in part because of their reproducibility [27]. These include fibronectins [28], DARPins [29], and ankyrins [30].

## 2. Non-Natural Nucleotides in Functional Nucleic Acids (DNA, RNA, Collectively xNA)

Even at the time when the concept of aptamers arose, it was clear that aptamers based on DNA and RNA might encounter functional limitations. The early authors fully recognized that the range of functional groups on (and therefore, presumably, the range of functionality enabled by) DNA and RNA molecules made of only four building blocks was limited compared with their protein counterparts. 

Therefore, as early as the 1990s, many groups attempted to increase the functionality of nucleic acids by adding functional groups to the already existing four building blocks [31,32,33,34] (for comprehensive reviews on aptamer developments [35,36,37]). These started with the simple hydrophobic groups introduced by Eaton et al., the non-covalent bound functionality of the cationic groups introduced by Benner et al., who sought to expand the coordination properties of the system, and others. By the end of the last decade, David Perrin et al. had added a different functional group to each of the four standard nucleotides, and found evidence for substantially improved catalytic activity [38,39]. Scott Silverman likewise added functionality to DNA libraries, eventually reporting amide bond cleavage [40,41]. This last case is historically remarkable, as several groups in this field had previously sought after sequence-specific peptide-cleaving aptazymes [42,43,44,45,46]. 

However, simply adding functional groups to standard nucleotides does not meet all of the challenges that obstruct DNA and RNA as sources for useful binding and catalytic molecules. With just four building blocks and two nucleobase pairs, DNA molecules have difficulty avoiding alternative folds that have similar energy. This leads to folding ambiguity, and likely allows non-functional folds to compete with functional folds in these species. This limitation of xNA as a scaffold for catalysis was quantitatively analyzed with respect to a Breaker–Joyce DNA aptazyme [47]. Over half of the low performance of that aptazyme as a catalyst was attributed to alternative, slowly interconverting, inactive folds that competed with the active fold [48].

The misfolding of xNA is also seen in nature, due to the low information density of a biopolymer built from just four building blocks. Often, this misfolding is associated with its own disease spectrum, such as for example with misfolded mitochondrial tRNA [49]. Indeed, exemplifying the “conformational hell” (a term from Uhlenbeck) displayed by RNA and its low information density [50,51], mitochondrial tRNA made from in vitro transcription usually does not fold into its native structures [52]. Simply adding functionality while keeping the same number of building blocks does not mitigate this problem.

Further, over functionalized xNA creates issues, as where every nucleobase has a functional group. Such molecules no longer behave similar to xNA if each nucleotide has its own tag [53], especially if it is hydrophobic, and they no longer need to follow Watson–Crick pairing rules. As a consequence, xNA molecules with functionality on each base are difficult to make with polymerases [54,55,56].

An alternative to adding functionality to existing nucleotides is to create new building blocks altogether and add them to the existing xNA pool. This approach has been taken on by the Benner laboratory and a few other groups in the past decade or so, such as the Hirao and Romesberg groups [57,58]. Hirao’s Ds–Px system is especially promising for the delivery of high-affinity binders [59], although its in vitro selection procedure does not yet allow for full randomization of the synthetic extra nucleotides in the starting library. 

## 3. The Artificially Expanded Genetic Information System (AEGIS)

This history describes a wish list among those who seek a directly evolvable system that delivers molecules that bind and catalyze on demand. Ideally, this system:

- Is designed, using the best chemical theory, to include functional groups that intrinsically deliver the desired reactivity. Given the limitations of current chemical theory, this design can be only “coarse” [60,61]; chemical theory is not adequate to “finely tune” those properties. However, and inaccessible to natural biopolymers, the functional groups need not be constrained to those that have been delivered to us by prebiotic chemistry and natural history; should chemical theory direct, they may include groups that manage the intrinsic difficulties of binding difficult targets or catalyzing difficult reactions, such as cleaving peptide bonds.
- Supports laboratory Darwinism, in order to allow the power of Darwinism to “finely tune” molecular systems to convert poor binders or catalysts into good ones. Darwinism cannot act prospectively. Therefore, it cannot anticipate what functional groups will be needed to solve future problems in binding and catalysis. Thus, the limits of Darwinism (it is a bad innovator, but an excellent refiner) complement the limits of design (a good innovator, but a bad refiner).
- Has added building blocks to ensure unique folds, but where:
- Only some of the building blocks carry functional groups to avoid “over decoration”.

**Figure 1:**
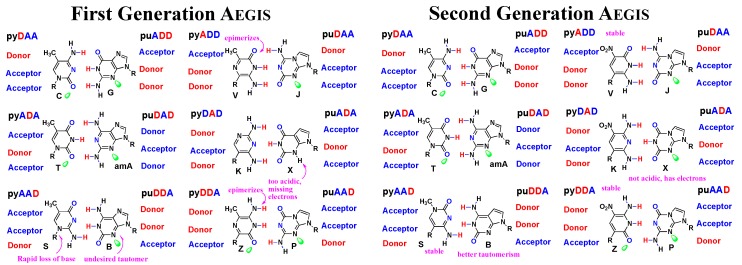
Examples of artificially expanded genetic information system (AEGIS) xNA building blocks in their “first generation” forms (left) and their “second generation” forms (right). AEGIS exploits the two rules that guide standard Watson–Crick nucleobase pairing: (a) size (large purines with small pyrimidines) and (b) hydrogen bonding (hydrogen bond acceptors A, with hydrogen bond donors D). Rearranging A and D groups gives an artificially expanded genetic information system (AEGIS) with up to 12 nucleotides forming six orthogonal pairs, with a functionality not present in standard DNA. AEGIS Z, for example, carries a nitro group (enhance the intrinsic binding potential of AEGIS libraries, and allowing the nucleobase to act as a general acid-base).

With these motivations, the Benner group developed parts of an artificially expanded genetic information system (AEGIS, Figure 1) [62]. AEGIS is a biopolymer similar to DNA [62,63,64], but with 12 building blocks, which in turn creates the opportunity to attach a number of functional groups that might be useful in binding and even catalysis. Figure 1 shows some examples of what has been made. Especially noteworthy is the use of the nitro group, which is a polyvalent general binder (remembering for example the ability of nitrocellulose to bind biomolecules). 

AEGIS nucleotides complete the Watson–Crick pairing concept by shuffling hydrogen bond donor and acceptor groups, forming additional orthogonal nucleobase pairs. They resemble natural nucleotides in size, shape, and pairing geometries, are independently replicable, and they do not interfere with DNA double helix structures that are fundamental for the recognition of the polymer by many natural enzymes. Most importantly, AEGIS nucleotides increase the information density of a nucleic acid molecule of a given length, and have the potential to increase functionality.

### 3.1. First and Second Generation AEGIS

We were not the first to hypothesize that an expanded genetic alphabet might be obtained by shuffling hydrogen bonding units. Alex Rich, some 20 years earlier, had recognized that isoguanine (a natural product) and isocytosine might possibly form a third pair [3] (the first generation S:B pair in Figure 1). Independently, Geoffrey Zubay proposed another alternative pair [65], not recognizing that his hypothetical structure for the small component lacked the aromatic planar geometry that the Watson–Crick model suggested was necessary for nucleobase stacking. Even the writer of ET. The Extraterrestrial understood the possibility; ET has DNA built from six nucleotides, “inosine and a pyrimidine we cannot identify” (Mathison, 1982).

However, synthesis as an activity was necessary to determine whether nucleobase pairing was as simple as the Watson–Crick model implied. In this undertaking, it soon became clear that more than one heterocyclic system would support, or “implement”, any particular nucleic acid hydrogen bonding pattern. For example, among natural nucleobases, uridine and pseudouridine both present a acceptor–donor–acceptor hydrogen bonding pattern. Those seeking to meet the grand challenge of creating an artificial genetic system needed to decide which heterocyclic system to synthesize to implement each of the orthogonal hydrogen bonding patterns.

In many cases, the first heterocyclic system that was prepared to implement each of the four additional hydrogen bonding patterns turned out not to be the best heterocycle to support genetics. Several first-generation AEGIS components suffered from chemical defects, which are indicated in magenta in Figure 1. For example, the pyrazine heterocycles that were first used to implement the pyADD and pyDDA hydrogen bonding patterns epimerized rapidly [66,67,68,69,70,71]. The purine ring system used to implement the puDDA hydrogen bonding patterns had a substantial amount of a minor tautomer that created nucleobase pairing ambiguity [72,73]. In a long process documented in the literature, second generation implementations of various bonding patterns were then synthesized to fix problems in the new DNA. 

These second generation improvements are summarized in Figure 1, in the right panel. The effort produced much new knowledge in heterocyclic chemistry. We applied chemical synthesis to create several generations of heterocycles that implemented the AEGIS concept on heterocycles with increasingly improved chemical stabilities [74], tautomeric ratios [75,76,77], study melting temperatures, and duplex stability [77]. Within the framework of the Watson–Crick pair, a rather comprehensive view of what heterocycles are possible in genetic systems emerged. 

### 3.2. The Structural and Molecular Biology of AEGIS

The development of AEGIS was supported by synthetic chemistry [78], mechanistic chemistry [79], enzymology [80], protein engineering [81], structural biology [82,83,84], and natural Darwinism [85,86,87,88,89,90]. Here, these worked together to not only improve AEGIS, but also to lead to a deeper understanding of how natural genetic biopolymers work.

For example, it seems unusual to those familiar with the design of artificial molecular recognition systems to have genetic information entrusted to flexible molecules. Most exercises in molecular recognition seek rigid size complementarity in the binder and the bindee. AEGIS allowed us to explore the flexibility of the DNA backbone and, by implication, its ability to enforce size complementarity. For example, a pair between two small nucleobases (a small:small pair) could stabilize the duplex, if they were joined by three hydrogen bonds, but less than a size complementary small:large pair. Indeed, a small:small pair joined by three hydrogen bonds stabilized the duplex as much as a small:large pair joined by just two hydrogen bonds [91]. 

**Figure 2:**
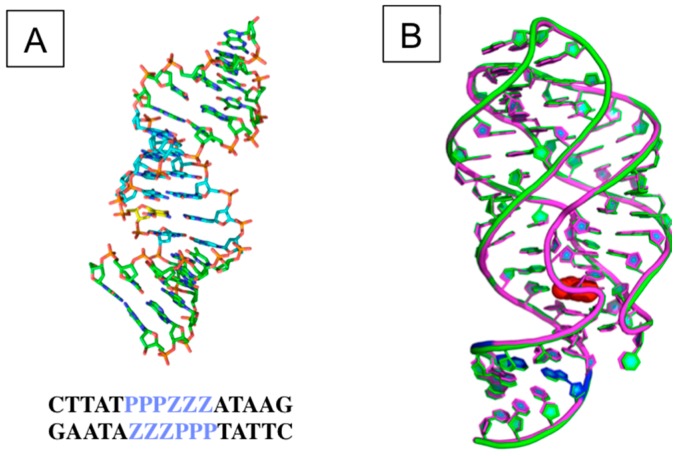
Crystal structures with Z:P AEGIS pairs: (**left**) A 16-mer duplex with six consecutive Z:P pairs [82]; (**right**) A single Z:P pair containing a RNA guanine riboswitch superimposed to its natural counterpart [92].

These biophysical studies were followed by crystallographic studies that showed that AEGIS components do, in fact, pair with Watson–Crick geometry. For example, the introduction of a single Z:P pair into the stem of a riboswitch marginally increased the stability of that stem; a crystal structure showed essentially no geometric perturbation [92] (Figure 2, right). In DNA, the crystal structure of a single Z:P pair likewise showed no substantial deviation from Watson–Crick geometry [93]. Indeed, duplexes with two or six Z:P pairs retain their overall Watson–Crick geometry [82,84] (Figure 2, left). 

With these tools in hand, we created a molecular biology to support laboratory in vitro evolution (LIVE) using AEGIS components. This includes in particular polymerases that are able to replicate AEGIS DNA. This challenge was approached two ways: by screening and optimizing commercially available polymerases [94,95], and by engineering polymerases by directed evolution to incorporate non-standard nucleotides [80]. Although neither approach produced enzymes or protocols that were absolutely immune to AEGIS losses and/or AEGIS/standard DNA mismatches (see the sections below), these studies set the stage for the development of laboratory in vitro evolution experiments with AEGIS libraries (AEGIS-LIVE). RNA polymerases [96] and reverse transcriptases [97] that interconvert AEGIS DNA and RNA, and restriction enzymes that cut specifically around AEGIS DNA [98] were also developed.

Moreover, we established analytical chemistry to support AEGIS-LIVE, including tools to analyze folding in functional AEGIS molecules (Circular Dichroism, X-ray crystallography) [85], and computational platforms to model the fold of functional AEGIS molecules based on biophysical studies of AEGIS DNA [77]. Of particular importance was the development of a strategy to convert AEGIS DNA to standard DNA in a traceable manner, which allows for AEGIS high-throughput sequencing [95,99]. This method takes advantage of the ability of polymerases to mismatch AEGIS nucleotides with standard nucleotides when the firsts are missing from the amplification mixture. Removing one AEGIS nucleotide at a time in parallel amplification reactions of the same target produces a “mismatch pattern” that allows for the identification of the type and location of the AEGIS nucleotide in the original sequence. 

At this point, the stage was set to attempt experiments that applied laboratory Darwinism to create AegisBodies, which are DNA-like molecules built from expanded genetic systems that we hoped would parallel protein antibodies, but without requiring the animal.

## 4. AEGIS Laboratory In Vitro Evolution (AEGIS-LIVE)

### 4.1. Cell-SELEX (Sistematic Evolution of Ligands by Exponential Enrichment) 

With a synthetic and molecular biology in place for AEGIS systems, our laboratory, in collaboration with the Tan laboratory at the University of Florida, attempted the first AEGIS-based in vitro evolution experiment in 2013, where a line of breast cancer cells, MDA-MB-231, was targeted in a Cell-SELEX experiment [100].

The library used in this selection contained a 20-nt long random region composed of ACTGZP nucleotides, and surrounded by primer binding sites made of standard DNA. This library was subjected to 12 rounds of selection that gradually reduced the number of cells and incubation times per cycle in order to increase the selection pressure. The surviving pool of cycle 12 was subjected to AEGIS to standard DNA conversion, and submitted for high-throughput sequencing. 

The most interesting results from this first AEGIS-LIVE experiment were that: (1) binders appeared after 9-12 rounds of selection, compared to the 15-20 rounds that have been reported as necessary to observe the growth of binders in previous Cell-SELEX experiments with standard nucleic acid libraries; and (2) the Z and P nucleotides, when present, were necessary for binding. The best aptamer from this selection, ZAP-2012, contained 1 P and 1 Z, which are both necessary for binding, and had a k_d_ of ~30 nM.

The second AEGIS-based Cell-SELEX, which was performed on HepG2 liver cancer cells, gave a little more information [93].

**Figure 3:**
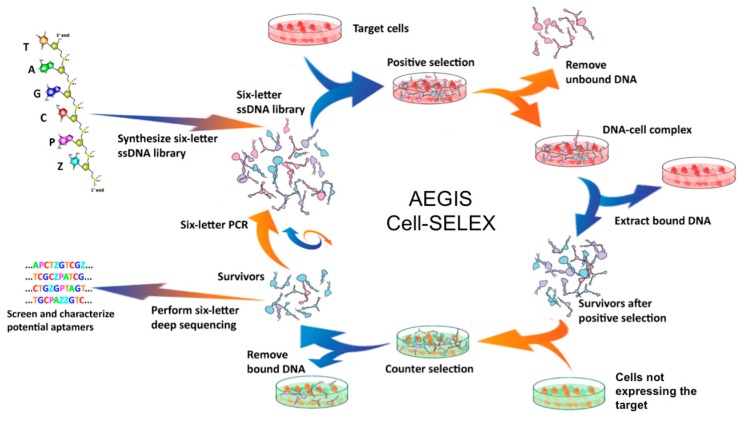
Schematic of AEGIS-Cell-SELEX (see text for details, adapted from [93]).

The schematic of this AEGIS-based Cell-SELEX is shown in Figure 3. In this case, the synthetic AEGIS-library contained 25 random nucleotides (ACTGZP), and a negative selection step was added to each cycle to increase specificity to the target cell line. Again, only 13 rounds of selection were needed to observe the growth of binders.

**Figure 4:**
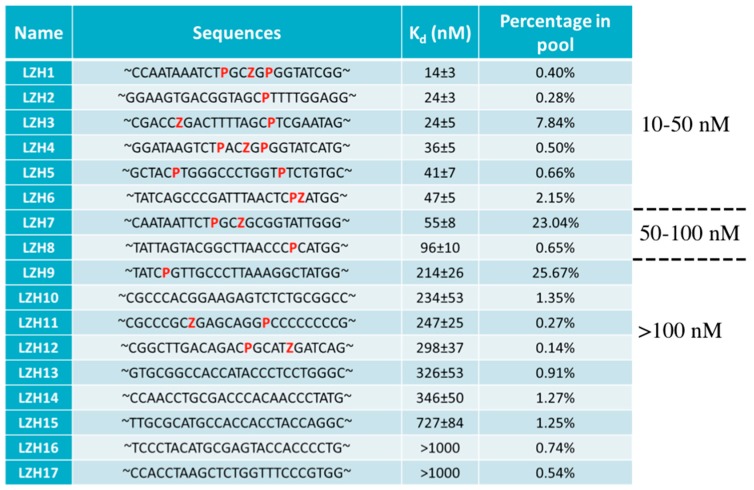
Sequences and k_d_ of aptamers evolved from AEGIS-Cell-SELEX against liver cancer cells. Percentage in the pool indicates the relative amounts of a particular sequence to the total sequences analyzed from cycle 13. AEGIS nucleotides are in red. Adapted from [93].

Several aptamers were obtained from deep sequencing, and a quantitative improvement in DNA performance with added nucleotides could definitely be seen. Indeed, the aptamers with the lowest k_d_ contained Z and/or P residues, and were as low as 14 nM, while ACTG-only aptamers had k_d_ ranging from 326 nM to more than 1 μM (Figure 4). Moreover, the aptamers were very specific for the cell line used during selection, and did not bind to other cell lines. This feature was not fully achieved in the first attempt at AEGIS-Cell-SELEX, where some aptamers also bound to some other cell lines.

In a third AEGIS-Cell-SELEX experiment, cell engineering was used to place glypican 3 (GPC3), a possible marker for liver cancer diagnostic, on the surface of a murine cell line that does not express GPC3 (1MEA). This engineered cell line (1MEA^hGPC3^) was used in positive selection cycles, while wild-type 1MEA cells were used in counter-selections [101]. In this case, the library’s randomized AEGIS region was 35 nt long. 

This experiment required only 11 rounds to produce populations showing substantial binding during bulk reactions. Many of the selected aptamers bound with various levels of specificity to GPC3; others appeared to bind to different proteins on the cell surface, perhaps newly emerging on the host cells as a consequence of their being engineered to express GPC3. Interestingly, the k_d_ for these aptamers were in the low to high nM range when tested on a hGPC3-positive human liver cancer cell line, HepG2. One aptamer in particular, LG5, which carried one Z residue but no Ps, showed a high level of specificity for the intended target regardless of the cell line expressing it, and had an apparent k_d_ (for HepG2) of 6 nM. Again, AEGIS nucleotides were found to be essential for binding. 

### 4.2. AEGIS-LIVE on Anthrax Protective Antigen

The first attempt to select for an AEGIS aptamer against an isolated, specific protein target was performed in 2016. Here, the AEGIS-LIVE experiment sought to obtain binders to the *Bacillus anthracis* 63-KDa version of protective antigen (PA63), the cleaved, heavier subunit of its precursor PA83 [102]. 

In this experiment, the six-letter ACTGZP AEGIS library contained only 25 randomized positions flanked by 15 nucleotide-long primer binding sites composed of standard DNA, with the aim of selecting for the smallest binders available in the ~10^14^ sampled different sequences (out of ~10^19^ total possible sequences). The target, PA63, was presented to the library immobilized to magnetic beads; binding oligonucleotides were recovered magnetically; and AEGIS-PCR with a single biotinylated primer was performed directly on survivors bound to the bead-coupled PA63. After 14 cycles of selection, the binder population had grown by ~30%, and the library from this cycle was subjected to transliteration and deep sequencing. One sequence named PA-Apt1, or PA1, dominated the surviving pool, with 96% of the total reads. This contained two P residues and, similar to the other five most represented sequences, no Z nucleotides, despite Z being present at 17% frequency in the starting library. 

**Figure 5:**
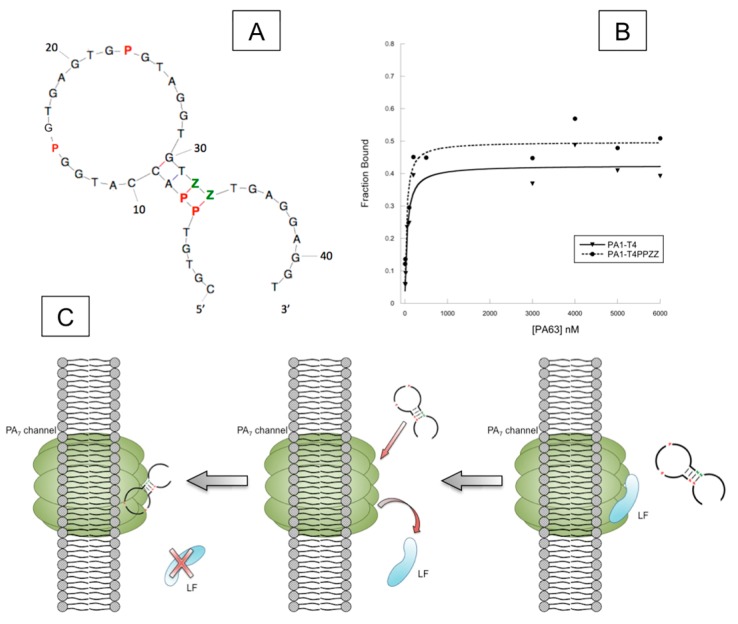
Anthrax protective antigen aptamer PA1T4PPZZ. (**A**) Aptamer’s predicted secondary structure and location of AEGIS nucleotides (in red, P, and green, Z). (**B**) Binding plots for PA1T4PPZZ and its parent molecule, PA1T4. (**C**) Graphical representation of an electrophysiology assay where PA63 in its channel conformation (green) is inserted into a membrane, and aptamer PA1T4PPZZ displaces the lethal factor (blue, LF) from its binding site on the channel.

In filter-binding assays, PA1 was found to be strictly specific for the 63-KDa version of protective antigen, and unable to bind to PA83, indicating that the PA1 epitope might at least in part reside on the portion of PA83 that becomes exposed after cleavage of the 20-KDa subunit. Extensive mutational and enzymatic analysis revealed that the two P were essential for binding to PA63, and that the folding of the molecule included a four base-pairs stem surmounted by a large highly compact and nuclease resistant loop (Figure 5A). While the parent molecule that was originally selected had a k_d_ of ~2.3 μM, subsequent truncations of the molecule reduced the binding constant to ~50 nM (PA1T4). Most interestingly, although expected from the structural studies mentioned above, when two base pairs in the stem were substituted with two P–Z pairs (PA1T4PPZZ), the molecule’s k_d_ lowered further to ~35 nM (Figure 5B). This is one example of the effectiveness of post-selection design, where a molecule’s features can be improved by the addition of strategically placed non-natural elements. 

In a stepwise type of analysis, AEGIS–PA63 aptamers were also tested against anthrax protective antigen biological activities. First, aptamers were found to bind to PA63 when this is already associated with its natural cell-receptor CMG2 (capillary morphogenesis protein 2, also known as ANTXR2), further narrowing the possible epitope of PA1 on its target surface, excluding the PA63 binding site to CMG2. Further, electrophysiology assays showed that PA1T4 competed with the lethal factor (LF) for binding to PA63 when this is assembled in its channel state across an in vitro membrane system (Figure 5C).

This molecule is the first example of an aptamer that has evolved towards a specific protein target from an expanded genetic alphabet using the molecular biology and analytical chemistry that has been developed to support it, but without the escamotages used in other systems to circumvent the limitations of extant biology. 

## 5. Conclusions

These results suggest promise, with much more work to be done. First, it is clear that AEGIS represents something new in the field of molecular recognition, and in synthetic biology. The “new” feature is its ability to evolve across what appears to be unconstrained sequence space. This is likely because the AEGIS pair still fits into the “aperiodic crystal structure” proposed by Schrödinger as being necessary to support Darwinism [103]. As the crystal data show, even if multiple AEGIS pairs are adjacent, the structure of the double helix survives. 

However, it is clear that polymerases that have evolved over billions of years to manage G:C and A:T pairs are not yet entirely “up to speed” when challenged to reproduce AEGIS DNA. Thus, while results from AEGIS-LIVE do not suggest a preference for certain regions of the expanded AEGIS sequence space, they do suggest a slow loss of AEGIS pairs during the PCR amplification that occurs between selection steps.

However, this also indirectly supports the hypothesis that added pairs and added functionality are valuable during the selection step. Thus, the best aptamers that these four experiments have delivered retain AEGIS Z or P (or both) despite the bias towards Z/P loss during the amplification step.

This has a parallel in standard LIVE with four nucleotide alphabets. Sequences rich in G appear to be favored in functional xNA molecules, which is likely because G can form a series of non-Watson–Crick interactions that allow the xNA molecules to fold. However, polymerases have difficulty replicating G-rich regions, causing PCR to be selectively biased against them. This fight between what is desired in the selection step, and what is tolerated in the amplification step in natural xNA may be a parallel to what we see with AEGIS alphabets.
